# Supplementary material for: The emergence of Omicron VOC and its rapid spread and persistence in the Western Amazon
Source: PLoS One. 2023 Aug 17;18(8):e0285742. doi: 10.1371/journal.pone.0285742 (PMC10434903; doi:10.1371/journal.pone.0285742)
Supplement: S1 Table — All genome sequences and associated metadata in this dataset are published in GISAID’s EpiCoV database. To view the contributors of each individual sequence with details such as accession number, Virus name, Collection date, Originating Lab and Submitting Lab and the list of Authors, visit 10.55876/gis8.230107pn. (PDF) [file pone.0285742.s001.pdf]

SUPPLEMENTAL TABLE

Data Availability

GISAID Identifier:	EPI_SET_230107pn
doi:	10.55876/gis8.230107pn

All genome sequences and associated metadata in this dataset are published in GISAID's EpiCoV database. To view the contributors of each individual sequence with details such as accession number, Virus name, Collection date, Originating Lab and Submitting Lab and the list of Authors, visit 10.55876/gis8.230107pn

Data Snapshot

- EPI_SET_230107pn is composed of 363 individual genome sequences.
- The collection dates range from 2019-12-24 to 2022-12-16;
- Data were collected in 2 countries and territories;
- All sequences in this dataset are compared relative to hCoV-19/Wuhan/WIV04/2019 (WIV04), the official reference sequence employed by GISAID (EPI_ISL_402124). Learn more at https://gisaid.org/WIV04.
