# Supplementary material for: The emergence of Omicron VOC and its rapid spread and persistence in the Western Amazon
Source: PLoS One. 2023 Aug 17;18(8):e0285742. doi: 10.1371/journal.pone.0285742 (PMC10434903; doi:10.1371/journal.pone.0285742)
Supplement: Attachment — Submitted filename: Response to Reviewers.docx [file pone.0285742.s002.docx]

**Requisitos adicionais**

1. Please ensure that your manuscript meets PLOS ONE's style requirements, including those for file naming. The PLOS ONE style templates can be found at

<https://journals.plos.org/plosone/s/file?id=wjVg/PLOSOne_formatting_sample_main_body.pdf> and <https://journals.plos.org/plosone/s/file?id=ba62/PLOSOne_formatting_sample_title_authors_affiliations.pdf>.

R: Manuscript was formatted in journal style.

2. Please provide additional details regarding participant consent. In the ethics statement in the Methods and online submission information, please ensure that you have specified (1) whether consent was informed and (2) what type you obtained (for instance, written or verbal, and if verbal, how it was documented and witnessed). If your study included minors, state whether you obtained consent from parents or guardians. If the need for consent was waived by the ethics committee, please include this information.

If you are reporting a retrospective study of medical records or archived samples, please ensure that you have discussed whether all data were fully anonymized before you accessed them and/or whether the IRB or ethics committee waived the requirement for informed consent. If patients provided informed written consent to have data from their medical records used in research, please include this information.

Changes made in line 69 to 74: “All experiments were conducted in accordance with the relevant guidelines and regulations and were exempted from the requirements for informed consent due to the pandemic timing making direct contact with the subjects impossible. In addition, the study poses no risk to subjects due to the use of RNA from naso- or oropharyngeal samples already collected and extracted for the diagnosis of COVID by the centers, with no need for additional collections”.

3. Thank you for stating the following financial disclosure:
 "This study was funded by Fundação Oswaldo Cruz de Rondônia – FIOCRUZ/RO, Departamento de Ciência e Tecnologia (DECIT), Fundação para o Desenvolvimento da Ação Científica e Tecnológica e à Pesquisa do Estado de Rondônia - FAPERO (Process:350.095.442.048.526.000.000/ 2016; Public bid invitation: 012/2016 PRO-RONDÔNIA and 001/2020 PPSUS) and by Instituto Nacional de Epidemiologia da Amazônia Ocidental - INCT EpiAmO. FGN is a CNPq fellow. Departamento de Ciência e Tecnologia (DECIT) of the Brazilian MoH, US/CDC and OPAS, Brazilian office."

Please state what role the funders took in the study. If the funders had no role, please state: "The funders had no role in study design, data collection and analysis, decision to publish, or preparation of the manuscript."
If this statement is not correct you must amend it as needed.
Please include this amended Role of Funder statement in your cover letter; we will change the online submission form on your behalf.

R: Changes made in line 315 to 317: The following sentence was added “ The funders had no role in study design, data collection and analysis, decision to publish, or preparation of the manuscript.”

4. Thank you for stating the following in the Acknowledgments Section of your manuscript:
 "The present study was developed by a group of researchers from Laboratório de Virologia Molecular da Fundação Oswaldo Cruz, in Rondônia, with financial support from the Genomic Coronavirus Fiocruz Network, Departamento de Ciencia e Tecnologia (DECIT), Fundação para o Desenvolvimento das Ações Científicas
e Tecnológicas da Pesquisa do Estado de Rondônia – FAPERO, Programa de Pesquisa para o SUS (PPSUS), as well as Instituto Nacional de Ciência e Tecnologia de
Epidemiologia da Amazônia Ocidental – INCT- EpiAmo who have been important contributors to scientific development in the Amazon region. Collaboration from
Coordenação de Aperfeiçoamento Pessoal de Nível Superior – CAPES, from whom some authors received financial aid (scholarships) during the production of this study, the Vice president of Vigilância em Saúde e Laboratórios de Referências of Fiocruz, Instituto de Biologia Molecular do Paraná (IBMP) and Laboratório Central de Saúde Pública de Rondônia (LACEN/RO) were essential for the development of the study"

We note that you have provided funding information that is not currently declared in your Funding Statement. However, funding information should not appear in the Acknowledgments section or other areas of your manuscript. We will only publish funding information present in the Funding Statement section of the online submission form.
Please remove any funding-related text from the manuscript and let us know how you would like to update your Funding Statement. Currently, your Funding Statement reads as follows:
 "This study was funded by Fundação Oswaldo Cruz de Rondônia – FIOCRUZ/RO, Departamento de Ciência e Tecnologia (DECIT), Fundação para o Desenvolvimento da Ação Científica e Tecnológica e à Pesquisa do Estado de Rondônia - FAPERO (Process:350.095.442.048.526.000.000/ 2016; Public bid invitation: 012/2016 PRO-RONDÔNIA and 001/2020 PPSUS) and by Instituto Nacional de Epidemiologia da Amazônia Ocidental - INCT EpiAmO. FGN is a CNPq fellow. Departamento de Ciência e Tecnologia (DECIT) of the Brazilian MoH, US/CDC and OPAS, Brazilian office."

Please include your amended statements within your cover letter; we will change the online submission form on your behalf.

R:The acknowledgements of the study's funding bodies have been removed, thus adjusting them to line 293 to 299: “The present study was developed by a group of researchers from Laboratório de Virologia Molecular da Fundação Oswaldo Cruz, in collaboration from Coordenação de Aperfeiçoamento Pessoal de Nível Superior – CAPES, from whom some authors received financial aid (scholarships) during the production of this study, the Vice president of Vigilância em Saúde e Laboratórios de Referências of Fiocruz, Instituto de Biologia Molecular do Paraná (IBMP) and Laboratório Central de Saúde Pública de Rondônia (LACEN/RO) were essential for the development of the study.”

R: Changes made in line 308 to 317: “Funding: This study was funded by Fundação Oswaldo Cruz de Rondônia - FIOCRUZ/RO, the Genomic Coronavirus Fiocruz Network, Department of Science and Technology (DECIT), Foundation for the Development of Scientific and Technological Action and Research of the State of Rondônia - FAPERO (Process:350. 095.442.048.526.000.000/ 2016; Public bid invitation: 012/2016 PRO-RONDÔNIA and 001/2020 PPSUS) and by Instituto Nacional de Epidemiologia da Amazônia Ocidental - INCT EpiAmO. FGN is a CNPq fellow. Department of Science and Technology (DECIT) of the Brazilian MoH, US/CDC and PAHO, Brazilian office. The funders had no role in study design, data collection and analysis, decision to publish, or preparation of the manuscript.”

5. Thank you for stating the following in the Competing Interests section:
 "At the time of submission, R.C.P.R, L.G.M and F.K.M were employees at IBMP, which manufactures and commercializes the test described in this study. The other authors declare no potential conflict of interest."

Please confirm that this does not alter your adherence to all PLOS ONE policies on sharing data and materials, by including the following statement: "This does not alter our adherence to PLOS ONE policies on sharing data and materials.” (as detailed online in our guide for authors <http://journals.plos.org/plosone/s/competing-interests>). If there are restrictions on sharing of data and/or materials, please state these. Please note that we cannot proceed with consideration of your article until this information has been declared.

Please include your updated Competing Interests statement in your cover letter; we will change the online submission form on your behalf.

R: Changes made in line 320 to 321: Inserting sentence at the end of the topic "This does not alter our adherence to PLOS ONE policies on sharing data and materials."

6. We note that you have stated that you will provide repository information for your data at acceptance. Should your manuscript be accepted for publication, we will hold it until you provide the relevant accession numbers or DOIs necessary to access your data. If you wish to make changes to your Data Availability statement, please describe these changes in your cover letter and we will update your Data Availability statement to reflect the information you provide.

The information that will be passed on comprises the sequence IDs collected in GISAID, which is already in the manuscript as background material (S1 Table) line 390 to 391.

7. We note that you have included the phrase “data not shown” in your manuscript. Unfortunately, this does not meet our data sharing requirements. PLOS does not permit references to inaccessible data. We require that authors provide all relevant data within the paper, Supporting Information files, or in an acceptable, public repository. Please add a citation to support this phrase or upload the data that corresponds with these findings to a stable repository (such as Figshare or Dryad) and provide and URLs, DOIs, or accession numbers that may be used to access these data. Or, if the data are not a core part of the research being presented in your study, we ask that you remove the phrase that refers to these data.

R: "Data not shown" referred to the map (Figure 1). With the removal of the map the article has all data according to PLOS policy.”

8. Your ethics statement should only appear in the Methods section of your manuscript. If your ethics statement is written in any section besides the Methods, please delete it from any other section.

R: Topic removed: “**Institutional Review Board Statement:** The project was evaluated and approved by the Research Ethics Committee of the Research Center for Tropical Medicine - CEPEM - Rondônia under protocol no. 4,000,086 and carried out in accordance with the ethical principles stipulated by the 1975 World Medical Assembly and the Ministry of Health (Resolution 466).”

9. We note that Figure 1 in your submission contain [map/satellite] images which may be copyrighted. All PLOS content is published under the Creative Commons Attribution License (CC BY 4.0), which means that the manuscript, images, and Supporting Information files will be freely available online, and any third party is permitted to access, download, copy, distribute, and use these materials in any way, even commercially, with proper attribution. For these reasons, we cannot publish previously copyrighted maps or satellite images created using proprietary data, such as Google software (Google Maps, Street View, and Earth). For more information, see our copyright guidelines: <http://journals.plos.org/plosone/s/licenses-and-copyright>.

We require you to either (a) present written permission from the copyright holder to publish these figures specifically under the CC BY 4.0 license, or (b) remove the figures from your submission:

a. You may seek permission from the original copyright holder of Figure 1 to publish the content specifically under the CC BY 4.0 license.

We recommend that you contact the original copyright holder with the Content Permission Form (<http://journals.plos.org/plosone/s/file?id=7c09/content-permission-form.pdf>) and the following text:
“I request permission for the open-access journal PLOS ONE to publish XXX under the Creative Commons Attribution License (CCAL) CC BY 4.0 (<http://creativecommons.org/licenses/by/4.0/>). Please be aware that this license allows unrestricted use and distribution, even commercially, by third parties. Please reply and provide explicit written permission to publish XXX under a CC BY license and complete the attached form.”

Please upload the completed Content Permission Form or other proof of granted permissions as an "Other" file with your submission.

In the figure caption of the copyrighted figure, please include the following text: “Reprinted from [ref] under a CC BY license, with permission from [name of publisher], original copyright [original copyright year].”

b. If you are unable to obtain permission from the original copyright holder to publish these figures under the CC BY 4.0 license or if the copyright holder’s requirements are incompatible with the CC BY 4.0 license, please either i) remove the figure or ii) supply a replacement figure that complies with the CC BY 4.0 license. Please check copyright information on all replacement figures and update the figure caption with source information. If applicable, please specify in the figure caption text when a figure is similar but not identical to the original image and is therefore for illustrative purposes only.
The following resources for replacing copyrighted map figures may be helpful:

USGS National Map Viewer (public domain): <http://viewer.nationalmap.gov/viewer/>
The Gateway to Astronaut Photography of Earth (public domain): <http://eol.jsc.nasa.gov/sseop/clickmap/>
Maps at the CIA (public domain): <https://www.cia.gov/library/publications/the-world-factbook/index.html> and <https://www.cia.gov/library/publications/cia-maps-publications/index.html>
NASA Earth Observatory (public domain): <http://earthobservatory.nasa.gov/>
Landsat: <http://landsat.visibleearth.nasa.gov/>
USGS EROS (Earth Resources Observatory and Science (EROS) Center) (public domain): [http://eros.usgs.gov/#](http://eros.usgs.gov/)
Natural Earth (public domain): <http://www.naturalearthdata.com/>

“It was not possible to obtain the Creative Commons Attribution License (CC BY 4.0), so we have chosen to remove the map at this point (figure 1).”

10. Please include captions for your Supporting Information files at the end of your manuscript, and update any in-text citations to match accordingly. Please see our Supporting Information guidelines for more information: <http://journals.plos.org/plosone/s/supporting-information>.

R: Changes made in line 480 to 484: **S1 Table. SARS-CoV-2 genomes.** All genome sequences and associated metadata in this dataset are published in GISAID’s EpiCoV database. To view the contributors of each individual sequence with details such as accession number, Virus name, Collection date, Originating Lab and Submitting Lab and the list of Authors, visit 10.55876/gis8.230107pn

11. Please review your reference list to ensure that it is complete and correct. If you have cited papers that have been retracted, please include the rationale for doing so in the manuscript text, or remove these references and replace them with relevant current references. Any changes to the reference list should be mentioned in the rebuttal letter that accompanies your revised manuscript. If you need to cite a retracted article, indicate the article’s retracted status in the References list and also include a citation and full reference for the retraction notice.

Two new references have been added to the methodology about the genome assembly, so that references 20 and 21 have been added to the following section in the methodology: “FASTQ reads were generated by the Illumina pipeline in BaseSpace. Consensus sequences were generated using DRAGEN COVID LINEAGE 3.5.1 to 3.5.3, according to the most updated version of this application in each sequencing run. Subsequently, the quality of the consensus files was analyzed using the Nextclade v1.5.2 tool [20], those with more than 1% "Ns" ambiguities had the FASTQ files imported into Geneious Prime 2021 for trimming and assembly using a custom workflow employing the BBDuk and BBMap tools (v38.84) and the NC_045512.2 RefSeq as a template with careful visual inspection. Using both approaches, we generated consensus sequences with average depth coverage greater than 800X, excluding duplicate reads. The genome-wide consensus sequences of SARS-CoV-2 were initially assigned to viral lineages using the Pango Lineage web application [21].”

Reviewers' comments:

Reviewer's Responses to Questions

**Comments to the Author**

Reviewer #1: This study reported the emergence of Omicron VOC and its rapid spread and persistence in the Western Amazon. It is a nice study with the clear methodology. There are a few comments:

1. What are subtypes of Omicron VOC?

Information found on the page 198-205: Twenty-seven Omicron VOC subtypes were found: “The analyses of the sequenced samples corresponded to 30.14% (167) BA.1; 18.05% (100) BA.1.1; 6.14% (34) BA.1.1.1; 0.18% (1) BA.1.1.14; 0.18% (1) BA.1.1.15; 0.18% (1) BA.1.1.18; 1.26% (7) BA.1.14; 12.82% (71) BA.1.14.1; 0.18% (1) BA.1.14.2; 0.90% (5) BA.1.15; 2.17% (12) BA.1.17.2; 0.18% (1) BA.1.20; 0.36% (2) BA.1.9; 2.35% (13) BA.2; 0.18% (1) BA.2.12.1; 0.18% (1) BA.2.36; 0.72% (4) BA.2.56; 0.18% (1) BA.2.81; 1.81% (10) BA.4; 0.18% (1) BA.4.1; 9.93% (55) BA.5.1; 0.18% (1) BA.5.1.15; 8.84% (49) BA.5.2.1; 0.36% (2) BA.5. 6; 0.54% (3) BE.9; 1.62% (9) BF.5 and 0.18% (1) BQ.1.1. To date, no cases of XBB.1.5 have been reported in the state of Rondônia.”

2. What CoVID-19 vaccines did the patients receive?

R: Changes made on pages 183 to 185: “The immunizers received by the study population were COVISHIELD, Sinovac, Comirnaty® | Pfizer Brazil, and Janssen-Cilag”.

3. How was the Illumina data analyzed to get complete genomes of SARS-CoV-2?

R: Changes made on pages 134 to 144: “FASTQ reads were generated by the Illumina pipeline in BaseSpace. Consensus sequences were generated using DRAGEN COVID LINEAGE 3.5.1 to 3.5.3, according to the most updated version of this application in each sequencing run. Subsequently, the quality of the consensus files was analyzed using the Nextclade v1.5.2 tool [20], those with more than 1% "Ns" ambiguities had the FASTQ files imported into Geneious Prime 2021 for trimming and assembly using a custom workflow employing the BBDuk and BBMap tools (v38.84) and the NC_045512.2 RefSeq as a template with careful visual inspection. Using both approaches, we generated consensus sequences with average depth coverage greater than 800X, excluding duplicate reads. The genome-wide consensus sequences of SARS-CoV-2 were initially assigned to viral lineages using the Pango Lineage web application [21].”

4. How was the phylogeny generated?

Initially, high quality reference sequences were retrieved from all the VOC Omicron lineages found in the study and then the dataset was aligned using MAFFT. The phylogeny was generated based on the maximum likelihood method. We used IQ-TREE 2 with the Ultrafast Bootstrap algorithm which is already frequently used for phylogenetic analysis applied to SARS-CoV-2.

Reviewer #2: Summary:
This manuscript describes the collection and basic analyses of the emergence and spread of SARS-CoV-2 variants in Rondônia, Brazil. This piece highlights the increase in SARS-CoV-2 sequencing over the last months in Rondônia, Brazil and presents substantial new country-specific genomic data which is important in understanding the evolutionary mechanisms, transmission, and infectivity of SARS-CoV-2 variants in Brazil.
Suggestions:
1. It’s quite unfortunate that phylogeographic analysis was not included in this study which would have been used to infer the geographic origin of the variants, the course of range expansions, and the presence of genetic bottlenecks. Can this aspect be incorporated into the study?

Unfortunately, to perform a robust analysis there are some limitations related to the insufficient number of high quality deposited sequences of particular lineages.

In addition, a long period of time will be required to run the analyses considering a large data set, which would not be possible for the proposed response period.

2. Fig 3 shows three (3) clusters of BA.1 in the tree. Any explanation for the topology incongruence?

The justification for this topology is that the BA.1 lineage is the common ancestor of other BA.1* lineages and widely dispersed in different locations.

This can be checked in: <https://nextstrain.org/ncov/gisaid/global/6m?tl=pango_lineage>

Moreover, the clusters were strongly supported by a satisfactory bootstrap value (>99).

3. Your references are not consistent. Kindly check the PLOS format.

The format of the references was revised according to the journal model.
